# Prestige bias in cultural evolutionary dynamics

**DOI:** 10.1098/rsos.230650

**Published:** 2024-07-10

**Authors:** Saar Egozi, Yoav Ram

**Affiliations:** ^1^ School of Computer Science, Reichman University, Herzliya, Israel; ^2^ School of Zoology, Faculty of Life Sciences, Tel Aviv University, Tel Aviv, Israel; ^3^ Sagol School of Neuroscience, Tel Aviv University, Tel Aviv, Israel

**Keywords:** prestige, influence, cultural transmission, cultural evolution, evolutionary model

## Abstract

If the traits of more successful individuals are more likely to be adopted, the resulting cultural transmission is described as success biased. In contrast, if the traits of ‘prestigious’ individuals—those who have already been copied many times—are more likely to be adopted, this is described as prestige-biased cultural transmission. In this case, prestige can be a convenient proxy for success. However, it is unclear how success and prestige biases interact to determine the outcome of cultural evolutionary dynamics. Here, we aim to clarify this using mathematical analysis and stochastic simulations. We find analytic approximations to the stochastic role-model choice process that facilitate the mathematical analysis and reduce the computational complexity of simulations. Approximations are given to the fixation probability and the fixation time of an invading cultural trait in different environments. Our results show that success bias effectively plays the role of natural selection, whereas prestige bias effectively plays the role of genetic drift. Prestige bias, which may be strong in highly social communities, also accelerates the evolutionary dynamics, as expected in a rich-get-richer process. These results signify a step forward in understanding how different cultural transmission biases interact.

## 1. Introduction

Cultural transmission of attitudes, preferences, beliefs, norms and behaviours may combine vertical transmission, in which parents transmit to their offspring; oblique transmission, in which adults (teachers, leaders and even strangers) transmit to unrelated offspring; and horizontal transmission, in which individuals from the same age cohort transmit to one another [1]. It has been demonstrated that non-vertical cultural transmission can maintain maladaptive traits, which can be beneficial in changing environments [2, 3].

Transmission biases may cause a cultural trait to have a higher rate of transmission than its frequency in the population. Success bias occurs when individuals prefer to copy from role-models that demonstrate success in some activity, such as fishing, growing yams, using medicinal plants [4] or hunting [5], and it can increase the probability of learning a trait that is present in those successful individuals [6]. Indeed, in a tournament between learning strategies [7], most winning strategies included a mixture of success-biased social learning and individual learning, implying that success-biased learning is a good strategy, but that by itself it is not enough to best other strategies, even when success is measured accurately. Jiménez & Mesoudi [8] also note that a way to acquire adaptive social information is by preferentially copying competent individuals within a valuable domain (which they also call success bias). However, they claim that competence within a domain is often difficult or impossible to directly assess, and therefore people tend to use indirect cues of success. Henrich & Broesch [4] also suggested that direct assessment of success may be ‘noisy, unreliable or unavailable’, and therefore copiers should also take into account indirect measures of perceived success (e.g. ‘great fishermen may be chosen as role-models for growing yams’.)

Boyd & Richerson [9, ch. 5] suggested that the assessment of success can be divided into three categories: direct bias, indirect bias and frequency-dependent bias. Direct bias occurs when one phenotype is more attractive than other phenotypes, and is evaluated by directly testing the trait. For example, an individual observing a ping-pong match can try the observed paddle grips to determine which grip is better. Frequency-dependent bias occurs when the probability of copying a phenotype is higher or lower than the frequency of the phenotype among demonstrators. For example, suppose the common paddle grip is used by 60% of the demonstrators; if this grip is adopted by 80% of copiers, then transmission is under positive frequency bias, also called conformity; if it is adopted by 40% of copiers, then transmission is under negative frequency bias or anti-conformity [10]. The effects of conformity and anti-conformity on cultural evolution have been studied with both models [11–13] and experiments [14]. Indirect bias occurs when a copier uses some observed phenotype to evaluate the attractiveness of a potential role-model. For example, an observer may copy the paddle grip of the ping-pong player who scored more points in the match, thus indirectly evaluating the grip by the points scored. However, this may cause mismatches between the copied trait and the rest of the cultural or genetic repertoire of the individual [15]. Furthermore, Boyd & Richerson [9, ch. 8] suggest that maladaptive traits may spread widely in a population if indirect bias is strong enough, e.g. by a runaway process caused by a cultural equivalent of sexual selection [16]. Indeed, helping behaviours can evolve owing to horizontal transmission bias even without any benefit to the recipient [17].

Henrich & Gil-White [18] noted that ‘the most skilled/knowledgeable role-models will, on average, end up with the biggest and most lavish clienteles, so the size and lavishness of a given model’s clientele size (the *prestige*) provides a convenient and reliable proxy for that person’s information quality’. Thus, they predicted that skilled individuals have higher status, that people preferentially copy high-status individuals, and therefore that prestigious individuals may be influential even beyond their domain of expertise. They defined prestige as ‘freely conferred deference’, in contrast to *dominance*, and provided examples from the anthropological literature [18]. Similarly, the New Oxford American Dictionary defines prestige as the ‘widespread respect and admiration felt for someone or something on the basis of a *perception* of their achievements or quality’. Chudek *et al*. [19] have also defined prestige bias as ‘a tendency to learn from individuals to whom others have preferentially attended, learned or deferred’ and demonstrated its occurrence in 3–4-year-old children. Nakata *et al*. [20] define such a bias as a ‘prestige bias based on second-order cues’, in which ‘social learners can use the extent of attention and the amount copied from others as cues for prestige’. Henrich & Broesch [4] have further suggested that prestige bias can, over generations, lead to cultural adaptations, and that although prestige can lead to maladaptive traits spreading in the population, it can also accelerate the spread of adaptive traits.

The distinction between success and prestige bias is important, as prestige is a context-dependent bias, rather than a content-dependent bias: it does not depend on the phenotype itself but rather on the number of copiers that have already copied each role-model, which may be easier and more accurate to estimate than success. Prestige bias is also frequency independent (refer to corollary 1), and thus it differs from conformity [11–13], which depends on the frequency of a trait in the population or in a sample of role-models, rather than the social dynamics of copying.

Prestige bias may be more common in humans than success bias [21]. In contemporary human society, social media make it especially easy to estimate the social and cultural influence individuals have over others, which can have an effect on decision-making. Online social networks such as *Facebook* and *Instagram* are known to affect the influence of individuals [22–24], and specific marketing practices have been invented to capitalize on this effect [25]. However, despite many mentions of prestige in the cultural evolution literature, there are few models of prestige bias.

Here, we develop a stochastic model of cultural transmission with both indirect success bias and prestige bias to examine their relationship in contribution to the cultural evolution of populations. We find analytic approximations for this model. We also find approximations for the probability and time to fixation of a ‘successful’ phenotype (i.e. subject to success bias) in both a constant and a periodically changing environment. Comparing these approximations to Kimura’s approximations for the fixation of a favourable allele [26,27], we demonstrate that success and prestige bias play the role of natural selection and genetic drift, respectively.

## 2. Models

We begin with a continuous-trait model with indirect success bias, previously suggested by Boyd & Richerson [9]. Note that the indirect success bias is owing to an indirect evaluation, in which a certain phenotype is used to evaluate the success of potential role-models. We extend this model to include prestige bias, which introduces a within-generation model-choice process. To facilitate mathematical analysis, we also develop a simpler version of the model with a dichotomous trait.

We implement our stochastic models and approximations, perform statistical analyses and produce figures using Python [28] with NumPy [29] and Matplotlib [30]. The source code is available on GitHub (https://github.com/yoavram-lab/PrestigeBias) and deposited on Zenodo [31].

### 2.1. Continuous trait

We follow the Boyd & Richerson model [9], assuming only oblique transmission of a single trait. This focus on oblique transmission (copying from non-parental adults) means that we can neglect fitness differences between trait values since there is no correlation between the traits of parents and offspring. We consider a population of N individuals, described by a single trait that takes continuous values. At each generation, N naive individuals, or copiers, each choose a single role-model from the entire previous generation. Each copier then copies its trait value from the chosen role-model. Note that our transmission models are slightly different from those modelled before [9,12,32], in which the population is infinite and each copier samples n role-models and then copies its trait from one or more of the sampled role-models.

Similar to a Wright–Fisher model, generations are non-overlapping, and the entire population is replaced in each generation. The population at time t can be described by $A(t)=\left(A_{1}(t),\ldots ,A_{N}(t)\right)$ where $A_{i}(t)$ is the trait value of individual i at time t, and the initial population is drawn from a standard normal distribution, A(0)~N(0,1). Cultural transmission is modelled by a function F such that


(2.1) $$A_{i}(t+1)=F_{i}(A(t))\;.$$

**Success bias.** Boyd and Richerson [9, ch. 8, pp. 247–249] describe a transmission algorithm by defining F, a weighted average of the traits of all role-models, as


(2.2) $$F_{i}(A)=\sum_{j=1}^{N}G_{i,j}\cdot A_{i,j}\;,$$

where $G_{i,j}$ is the success bias of role-model j in the eyes of copier i,


(2.3) $$G_{i,j}=\frac{\beta \left(A_{i,j}\right)}{\sum_{k=1}^{N}\beta \left(A_{i,k}\right)}\;,$$

$A_{i,j}$ is the absolute trait value that copier i estimates for role-model j with some error $e_{i}\sim N(0,\eta^{2})$,


(2.4) $$A_{i,j}=A_{j}+e_{i},$$

and β(⋅) is the bias function that quantifies the success bias of a role-model [9, eqn. 5.11],


(2.5) $$\beta (A_{i,j})=b\cdot \exp\left(-\frac{{\left(A_{i,j}-\hat{A}\right)}^{2}}{2J}\right)\;,$$

with $\hat{A}$ as the arbitrary optimal trait value and J and b as parameters that control the bias strength; unless otherwise mentioned, we set b=J=1. Therefore, $G_{i,j}$ is a relative success score that copier i assigns to role-model j.

Boyd & Richerson [9] note that the deterministic blended transmission algorithm they use has alternatives. We develop a similar stochastic model with transmission from a single random role-model where instead of equation (2.2) we define the transmission function F as a random variable with its distribution given by


(2.6) $$\mathrm{Pr}\left(F_{i}(A)=A_{j}\right)=G_{i,j}\;;$$

here $G_{i,j}$ is the probability that copier i chooses to copy the trait of role-model j.

**Prestige bias.** We introduce a new element to the model by assuming that in each generation copiers choose their role-models one by one so that the choice of one copier can affect the choice of other copiers. We formulate this assumption in the following. Denote by $K_{i,j}$ the number of copiers that chose role-model j after copier i chose a role-model. Thus, i out of N copiers had already chosen a role-model, $\sum_{j=1}^{N}K_{i,j}=i$, and there are N−i copiers that have yet to choose a role-model. The stochastic process of role-model choice,


(2.7) $${\left\{K_{i}=(K_{i,1},\ldots ,K_{i,N})\right\}}_{i=1}^{N}\;,$$

is described by the recurrence equation


(2.8) $$K_{i,j}=K_{i-1,j}+S_{i,j},\;\;\;\;i,j=1,2,\ldots ,N\;,$$

where $S_{i,j}=1$ if the ith copier chose role-model j and 0 otherwise, and the initial state is $K_{0,j}=0$.

Following equation (2.6), the probability that the ith copier chose role-model j is given by the *influence* of role-model j in the eyes of copier i,


(2.9) $$Pr(S_{i,j}=1|S_{1,j},S_{2,j},...,S_{i-1,j})=G_{i,j}\;.$$

The influence $G_{i,j}$ of role-model j in the eyes of copier i is determined by success—the estimated biased trait value $\beta (A_{i,j})$—and prestige—the number of copiers that chose role-model j before copier i, $K_{i-1,j}$, replacing equation (2.3) with


(2.10) $$G_{i,j}=\frac{\alpha_{ij}\cdot \beta (A_{i,j})+(1-\alpha_{ij})\cdot K_{i-1,j}}{W_{i}}\;,$$

where $W_{i}$ is a normalizing factor that sums the numerator over all role-models (1≤j≤N) to ensure $\sum_{j=1}^{N}G_{i,j}=1$. Here, the success-bias weight $\alpha_{i,j}$ determines the relative weighting of success and prestige bias. It is a characteristic of the interaction of role-model j with copier i that determines the relative significance of success versus prestige bias in the role-model’s overall influence in the eyes of the copier. Different individuals may evaluate the importance of success and prestige differently. Additionally, we assume each role-model displays its prestige and success individually. For example, individuals with more followers but lacking skill may emphasize the number of their followers rather than their skill (i.e. have lower $\alpha_{i,j}$ value). Finally, the trait of role-model j estimated by copier i, $A_{i,j}$, remains as in equation (2.4).

### 2.2. Dichotomous trait

We introduce a simplified version of the above model where the trait has only two phenotypes: an optimal phenotype $\hat{A}$ and a suboptimal phenotype A. All role-models with the same phenotype will contribute to the probability that phenotype is transmitted and thus prestige is determined by the number of copiers that have already chosen a role-model with either phenotype. In addition, for simplicity and for easier mathematical analysis, we assume α is homogeneous and constant ($\alpha_{i,j}=\alpha$), which entails exchangeability between role-models. Therefore, the probability that the ith copier copies phenotype A is


(2.11) $$G_{i,A}=\frac{\alpha \cdot (N-X)\beta (A)+(1-\alpha )\cdot K_{i-1,A}}{\alpha \cdot (N-X)\beta (A)+\alpha \cdot X+(1-\alpha )\cdot (i-1)}\;,$$

where X is the number of role-models with trait $\hat{A}$ and $K_{i-1,A}$ is the number of copiers that already chose A when copier i chooses a role-model, and assuming that $\beta (\hat{A})=1$ (thus the term αX in the denominator). Complementing this, the probability of the ith copier to copy phenotype $\hat{A}$ is $G_{i,\hat{A}}=1-G_{i,A}$. Electronic supplementary material, figure S1 shows some examples of model dynamics for various values of α.

## 3. Results

Our models are defined by two nested stochastic processes. Change over multiple generations is described by the dynamics of the phenotype distribution at each generation, ${\left\{A(t)\right\}}_{t}$ (refer to equation (2.1)). The transition from one generation to the next is described by the number of copiers each role-model has after i copiers have chosen a role-model, ${\left\{K_{i}\right\}}_{i=1}^{N}$ (refer to equation (2.7)). We emphasize that the models are nested: A(t+1) can be computed from A(t) by evaluating $K_{N}$, where $K_{N,j}$ is the number of copiers that chose role-model j after all copiers chose a role-model. However, the former requires iterating over equations (2.8) and (2.9). Thus, we sought to find an equivalent stochastic process that has the same joint distribution as $K_{N}$. We found two approximations for the distribution of $K_{N}$, summarized here and explained in detail below. In both approximations, we assume that the success-bias weight is either completely homogeneous, $\alpha_{i,j}=\alpha$, or that $\alpha_{i,j}=\alpha_{j}$ is a characteristic of role-model j that does not vary between copiers. Note that these approximations apply for both the continuous-trait (equation (2.10)) and the dichotomous-trait (equation (2.11)) models.

**Generalized binomial distribution approximation (GBD).** The number of copiers of a specific role-model at each step, $K_{i,j}$, follows the *generalized binomial distribution* [33] and, therefore, (i) the expected number of copiers of role-model j equals its influence in the eyes of the first copier, multiplied by the total number of copiers, that is, $E[K_{N,j}]=N\cdot G_{1,j}$ if trait estimation error is uniform for all copiers ($e=e_{i}$ for i=1,…,N); and (ii) the expected number of copiers of each role-model equals its relative biased trait value, similar to the role of relative fitness in population-genetic models, that is, $E[K_{N,j}]=\beta (A_{j}+e)/\bar{\beta }$ if the bias weight is uniform for all role-models ($\alpha =\alpha_{j}$ for j=1,…,N), where $\bar{\beta }=1/N\sum_{j=1}^{N}\beta (A_{j}+e)$ is the population mean estimated trait value.

**Dirichlet-multinomial distribution approximation (DMD).** The role-model choice process, ${\left\{K_{i}\right\}}_{i=1}^{N}$, is equivalent to a *Pólya urn* model if trait estimation error is uniform for all copiers ($e=e_{i}$ for all i=1,…,N). Hence, the number of copiers of each role-model $K_{N}$ at the end of the role-model choice process follows the DMD.

After finding these approximations for the role-model choice process, we focus on the dichotomous-trait model, in which mathematical analysis is simpler, and study the fixation probability and time in both a constant and a changing environment.

### 3.1. Generalized binomial distribution approximation

The GBD emerges from a series of dependent Bernoulli trials (in contrast to the standard binomial distribution in which trials are independent) and is denoted by GenBin(n,p,θ) where n is the number of trials, p is the probability of success of the first trial and θ is the correlation between trials (θ can be estimated from data, but its value is insignificant for our approximation). Note that θ=0 gives the standard binomial distribution.

***Result 3.1*** (*GBD approximation*). *The number of copiers of role-model* j *after* i *copiers have chosen a role-model follows the generalized binomial distribution,*


$$K_{i,j}\;∼\;GenBin\left(i,\alpha_{j}\cdot \beta \left(A_{j}+e\right),\theta \right),$$

*if* $e_{i}=e$ *for all copiers* i=1,…,N*; the success-bias weight only depends on the role-model and not the copier, i.e.* $\alpha_{i,j}=\alpha_{j}$ *for all* i=1,…,N*; and* θ *is the correlation between successive role-model choices.*

*Proof*. Let $Q_{j}(k,i)=P(K_{i,j}=k|K_{i-1,j})$ be the probability that exactly k out of i copiers choose role-model j given $K_{i-1,j}$ out of i−1 copiers chose role-model j. Using conditional probability and equation (2.8),


(3.1) $$Q_{j}(k,i)=P_{j}(S_{i,j}=1|k-1,i-1)\cdot Q_{j}(k-1,i-1)+P_{j}(S_{i,j}=0|k,i-1)\cdot Q_{j}(k,i-1)\;,$$

where $S_{i,j}=1$ when the ith copier chooses role-model j. Equation (3.1) is equivalent to eqn (2.1) in Drezner & Farnum [33], which completes the proof.

This result gives the following corollary on the expected number of followers of a given role-model j by the end of the role-model choice process, $K_{N,j}$.

***Corollary 3.1.*** *The expected number of copiers of role-model* j *after all copiers have chosen a role-model is* $E[K_{N,j}]=N\cdot G_{1,j}$*, where* $G_{1,j}$ *is the probability of the first copier to copy role-model* j*. In addition,* $E[K_{N,j}]=\alpha_{j}\cdot \beta (A_{j}+e)/\bar{\alpha \cdot \beta (A+e)}$*, where the averaging in the denominator is over the role-models index,* j*.*

*Proof.* The expected value of the GBD is $E[K_{N,j}]=N\cdot Q_{j}(1,1)$, refer to Drezner & Farnum [33, eqn. 2.3]. Here, $Q_{j}(1,1)$ is the initial probability to choose role-model j, before any role-model choices are made, such that $Q_{j}(1,1)=G_{1,j}$ by definition. The rest of the proof is in appendix A.

Note that $G_{1,j}=\alpha_{j}\beta \left(A_{j}\right)/\sum_{i=1}^{N}\alpha_{j}\beta \left(A_{j}\right)$ (refer to equation (2.10) with $K_{0,j}=0$). In the limit of $\alpha_{j}\to 0$, that is, with only prestige bias, we get $G_{1,j}=1/N$, and from corollary 1, the expected number of copiers of role-model j is 1. Therefore, prestige bias is frequency independent, in contrast to conformity bias.

The special case where the bias weight is uniform for all role-models ($\alpha =\alpha_{j}$ for j=1,…,N) is interesting, because we can evaluate the expected number of copiers using a linear equation,


(3.2) $$E[K_{N,j}]=N\cdot \frac{\alpha \cdot \beta (A_{j}+e)}{\sum_{m=1}^{N}\alpha \cdot \beta (A_{m}+e)}=\beta (A_{j}+e)/\bar{\beta (A+e)}\;,$$

where the only variable is $A_{j}+e$, because $\bar{\beta (A+e)}$ is the mean of the distribution of the trait values, modified by some constant parameters of β. We can then write $L=1/\bar{\beta (A+e)}$ and


(3.3) $$E[K_{N,j}]=L\cdot \beta (A_{j}+e)\;.$$

**Numerical validation.** To validate that the GBD approximation for the number of copiers of a role-model is correct (equation (3.2)), we ran 1000 simulations of the full model, and compared the results with corollary 1. We compare the distribution of the number of copiers by plotting the histograms of both our simulation results and the expected values based on corollary 1.

Although basic, electronic supplementary material, figure S2 shows a good fit of the GBD approximation. We perform more extensive validations on the DMD (see below), because that is what we will use in our analysis.

### 3.2. Dirichlet-multinomial distribution approximation

**Pólya urn model.** This stochastic process consists of N draws from an urn with an initial number of coloured balls of M colours. When a ball is drawn, it is then placed back in the urn together with an additional new ball of the same colour. Let $U_{i}=\{u_{i,1},u_{i,2},...,u_{i,M}\}$ where $u_{i,j}$ is the number of balls of the jth colour in the urn after i draws. Let $S_{i,j}=1$ when drawing a j-coloured ball on the ith draw and 0 otherwise. The probability that $S_{i,j}=1$ given $U_{i-1}$ is


(3.4) $$P(S_{i,j}=1|U_{i-1})=\frac{u_{i-1,j}}{\sum_{m=1}^{M}u_{i-1,m}}=\frac{o_{j}+w_{i-1,j}}{\sum_{m=1}^{M}o_{m}+w_{i-1,m}}=\frac{o_{j}+w_{i-1,j}}{i-1+\sum_{m=1}^{M}o_{m}}\;,$$

where $o_{j}$ is the initial number of balls of colour j in the urn and $w_{i,j}$ is the cumulative number of new balls that were added to the urn after i draws of colour j.

***Result 3.2.*** (*Pólya urn model*). *The role-model choice process,* ${\left\{K_{i}\right\}}_{i=1}^{N}$, *is equivalent to a Pólya urn model if both the trait estimation error and the success-bias weight are constant in the population,* $e_{i}=e$ *for all* i=1,…,N *and* $\alpha_{i,j}=\alpha$ *for all* i,j=1,…,N.

*Proof*. Write $\alpha^{\prime }=\frac{\alpha }{1-\alpha }$ as the success-bias weight ratio, and $A_{j}^{\prime }=A_{j}+e$. From equation (2.10) and because $\sum_{j=1}^{N}K_{i,j}=i$, we have


(3.5) $$G_{i,j}=\frac{\alpha^{\prime }\beta (A_{j}^{\prime })+K_{i-1,j}}{\sum_{m=1}^{N}\left[\alpha^{\prime }\beta (A_{m}^{\prime })+K_{i-1,m}\right]}=\frac{\alpha^{\prime }\beta (A_{j}^{\prime })+K_{i-1,j}}{i-1+\sum_{m=1}^{N}\alpha^{\prime }\beta (A_{m}^{\prime })}\;.$$

Substituting M=N, $o_{j}=\alpha^{\prime }\beta (A_{j}^{\prime })$ and $w_{i,j}=K_{i,j}$ in equation (3.4) gives equation (3.5), thus completing the proof.

Frigyik *et al*. [34, §2] prove that the proportion of different coloured balls in a Pólya urn model converges to the Dirichlet distribution as the number of draws (the population size, N, in our model) approaches infinity, based on the Martingale convergence theorem [35]. We therefore have an approximation for the relative influence each role-model has when evaluated by copiers. Thus, choosing the role-models for all copiers is equivalent to drawing from a multinomial distribution where the parameters are the modified weights from a Dirichlet distribution and we have the following corollary.

***Corollary 3.2*** (*Dirichlet-multinomial distribution approximation*). *The number of copiers of each role-model approximates a Dirichlet-multinomial distribution,* $K_{N}\sim DirMul(N,G_{1})$*, under the conditions of result 2.*

**Numerical validation.** We next validated the DMD approximation of our model and tested its sensitivity to the assumptions ($e_{i}=e$ and $\alpha_{i}=\alpha$ for i=1,…,N) by comparing the results of stochastic simulations of our model (equation (2.11)) with the DMD approximation (corollary 2). We used a relatively small population size, N=100, thus validating that the approximation is in good agreement even for small N, despite the assumption of large N in the proof by Frigyik *et al*. [34, §2]. First, we computed an observed distribution of the number of copiers from the average empirical distribution of multiple simulations. We then compared this observed distribution with the expected theoretical DMD (electronic supplementary material, figure S3A). The difference in distributions was not perceived when plotting both distributions on the same figure, so we used the difference instead. The maximum difference is 0.5 role-models, which indicates a very good fit. In addition, we tested the likelihood of the observed data being drawn from the DMD, against a shuffle of the parameters vector of the DMD itself (electronic supplementary material, figure S3B). We see that the negative log-likelihood of the observed data is much higher than any other shuffled version of the parameters vector, strongly supporting our approximation.

Next, we examined the fixation probability and fixation time of a ‘successful’ phenotype $\hat{A}$ (i.e. favoured by success bias) when invading a population of phenotype A and compared results from the full model and the DMD approximation. Thus, we assume the population has a single individual with phenotype $\hat{A}$ and N−1 individuals with phenotype A. We find that the number of simulations needed to sufficiently approximate our model with the DMD approximation is roughly 1000 (electronic supplementary material, figure S4). We examined the robustness of the DMD approximation to relaxing the following assumptions. First, we relaxed our assumption of constant estimation error e. Estimation error in the original model was drawn from a normal distribution and added to the trait value before the evaluation of the success bias ($A_{i,j}=A_{j}+e_{i}$). When estimation error is applied, we sample $e_{i}$ for each copier i from a normal distribution with an expected value 0 and variance $\eta^{2}$. Even when the estimation error variance is 0.1, both the fixation probability and fixation time DMD approximations are accurate (electronic supplementary material, figure S5). We also relaxed our assumption of a uniform bias weight α (i.e. $\alpha_{i}=\alpha$). We allowed α to vary in the population, drawing $\alpha_{j}$ for each role-model j from a normal distribution such that $\alpha_{j}\sim N(0.5,\epsilon )$ where ϵ is between $10^{-7}$ and $10^{-1}$. We found again that results of the DMD approximation are similar to those from stochastic simulations of the full model (electronic supplementary material, figure S6).

### 3.3. Fixation probability and time

After finding that the DMD is a good approximation of the (within-generation) role-model choice process, we turn our attention to the (between-generation) evolutionary dynamics. We focus on the fixation probability and conditional fixation time (conditioned on the population reaching fixation) of a ‘successful’ phenotype, using a diffusion-equation approximation approach, similar to analyses of population-genetic models [26,27,36]. We are mainly interested in the effect of the success-bias weight, α, which determines the relative effect of success and prestige bias, given by equation (2.10).

For simplicity, we use the dichotomous-trait model, which also assumes a constant success-bias weight $\alpha_{i,j}=\alpha$, and we do not include trait estimation error in this analysis, i.e. $e_{i}=0$. As shown above, transmission in our model is approximately DMD (corollary 2 and equation (3.5)). We focus on two scenarios: the first scenario is of a ‘constant environment’ in which the same phenotype, $\hat{A}$, is always favoured by success bias; the second scenario is of a ‘changing environment’ in which the phenotype favoured by success-bias cycles between the invading phenotype $\hat{A}$ and the resident phenotype A (i.e. $\hat{A}$ starts as the rare phenotype).

**Drift and diffusion terms in a constant environment.** We start by finding the expectation and variance of the change in frequency from one generation to the next, which are the drift and diffusion terms of the diffusion equation. Let x and $x^{\prime }$ be the frequency of phenotype $\hat{A}$ in a population with N individuals in the current and next generation, respectively. We set β to be the success bias of phenotype A relative to phenotype $\hat{A}$, such that $\beta =\beta \left(A\right)/\beta \left(\hat{A}\right)<1$. Then (refer to appendix B for derivation),


(3.6) $$\begin{array}{l} E\left[x^{\prime }-x\right]=x\left(1-x\right)\left(1-\beta \right)+o\left(1-\beta \right)\;,\\ V\left(x^{\prime }-x\right)=x\left(1-x\right)\left(\frac{1}{\alpha N+\left(1-\alpha \right)}\right)+o\left(\frac{1}{\alpha N+\left(1-\alpha \right)}\right)\;. \end{array}$$

This analysis gives an interesting result relating the parameters α and β to parameters of the classical Wright–Fisher model from population genetics: the selection coefficient s, a measure of the effect of natural selection on the change in frequency of genotypes and the effective population size, $N_{e}$, a measure of the effect of random genetic drift on the change in frequency of genotypes. In a diffusion-equation approximation of the classical Wright–Fisher model, the expectation and variance of the change in frequency are $E[x^{\prime }-x]=sx(1-x)+o(s)$ and $V[x^{\prime }-x]=x(1-x)/N_{e}$ [26, eqn. 7], respectively. Therefore, we have the following result.

***Result 3.3*** (*Effective selection coefficient and population size*). *The effective selection coefficient* s *and effective population size* $N_{e}$ *can be written in terms of the success coefficient* β *(**equation (2.5)**), the success-bias weight* α *(**equation (2.10)**) and the population size* N *as*


(3.7) $$s=1-\beta =\frac{\beta (\hat{A})-\beta (A)}{\beta (\hat{A})},\;\;\;\;N_{e}=\alpha N+(1-\alpha )\;.$$

Note that when N>>1, $N_{e}\approx \alpha N$, resulting in a very convenient expression.

Using our effective selection coefficient, s=1−β, and effective population size, $N_{e}$, with the population-genetics fixation probability approximation given by Kimura [26, eqn. 8], we obtain the following result.

***Result 3.4*** (*Fixation probability*). *The fixation probability of an invading phenotype favoured by success bias is approximately*


(3.8) $$\pi (x)=\frac{1-e^{-2(1-\beta )N_{e}x}}{1-e^{-2(1-\beta )N_{e}}},$$

*where* x *is the initial frequency of the invading phenotype.*

Similarly, we can use 1−β and $N_{e}$ in the population-genetics fixation time approximation given by Kimura & Ohta [27, eqn. 17].

***Result 3.5*** (*Fixation time*). *The expected fixation time (conditioned on fixation) from an initial frequency* x *is approximately*


(3.9) $$T(x)=J_{1}(x)+\frac{1-\pi (x)}{\pi (x)}\cdot J_{2}(x),$$

where $N_{e}=\alpha N+\left(1-\alpha \right),\;S=N_{e}\left(1-\beta \right),$ *and*


(3.10) $$\begin{array}{l} J_{1}\left(x\right)=\frac{-1}{\left(1-\beta \right)\left(e^{-2S}-1\right)}\int_{x}^{1}\frac{1-e^{2S\xi }-e^{-2S\left(1-\xi \right)}+e^{-2S}}{\xi \left(1-\xi \right)}d\xi \;,\\ J_{2}\left(x\right)=\frac{-1}{\left(1-\beta \right)\left(e^{-2S}-1\right)}\int_{0}^{x}\frac{\left(1-e^{2S\xi }\right)\left(e^{-2S\xi }-1\right)}{\xi \left(1-\xi \right)}d\xi \;. \end{array}$$

Note that these integrals cannot be solved in closed form, and are estimated numerically.

**Figure 1.**
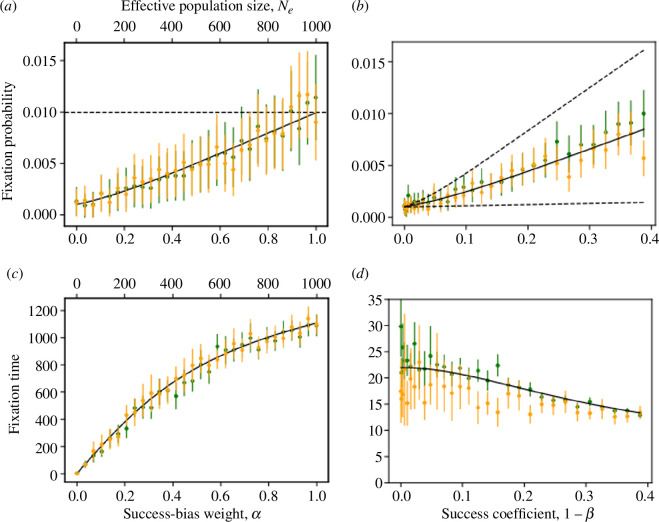
Fixation probability and time in a constant environment. The effect of the success-bias weight α (bottom *x*-axis) and effective population size, $N_{e}$ (top *x*-axis) on the fixation probability (*a*) and the conditional fixation time (*c*), and the effect of the success coefficient, or effective selection coefficient, 1−β, on the fixation probability (*b*) and the conditional fixation time (*d*). Our approximation (black; equation (3.8)) agrees with both DMD simulations (green) and Wright–Fisher simulations (orange). Panel (*a*) shows a dashed line at 2(1−β), which is reached by our approximation when α=1. Panel (*b*) has three lines: solid for our approximation for α=0.01 and dashed for α=0 (bottom) and α=0.02 (top). Markers are averages of 10000 simulations. Error bars show 95% confidence intervals. Here, population size is N=1000; panels (*a*) and (*b*): A=0.9 and $\hat{A}=1$ (1−β=s=0.005); panels (*c*) and (*d*): 0.01<A<0.99 and $\hat{A}=1$, which determines 1−β via $\beta =\beta (A)/\beta (\hat{A})$ and equation (2.5), α=0.01.

Results 4 and 5 lead to the following observations. First, the fixation probability increases (figure 1*b*) and the fixation time decreases (figure 1*d*) as a function of the success coefficient 1−β, which acts as an effective selection coefficient. Second, the fixation probability increases with the success-bias weight α (figure 1*a*), reaching a maximum at 2(1−β)=2s when there is no prestige bias (α=1), in which case the effective population size equals the actual population size (equation (3.7)). Third, and in contrast, the fixation time conditional on fixation is actually *shorter* with low values of α, that is, when prestige bias is strong (figure 1*d*). This is because prestige bias accelerates the evolutionary dynamics owing to a *rich-get-richer* process. Thus, when fixation occurs with strong prestige bias, it occurs faster than it does with strong success bias.

**Numerical validation.** We compare our approximations (equations (3.8) and (3.9)) with results of simulations of our dichotomous model using various α and β values, as well as simulations of the Wright–Fisher model, using the effective selection coefficient, 1−β, and effective population size, $N_{e}=\alpha N+(1-\alpha )$. We find that the two models have similar dynamics, and both are well approximated by our approximations (figure 1).

**Changing environment.** After finding a good approximation in a constant environment, where the ‘successful’ trait is always $\hat{A}$, we proceeded to find an approximation for a periodically changing environment. Following Ram *et al*. [2], we denote k as the number of generations in which the invading phenotype is favoured by success bias, and l as the number of generations in which the resident phenotype is favoured by success bias. Thus, during the first k generations of the environmental cycle, $\beta =\frac{\beta \left(A\right)}{\beta \left(\hat{A}\right)}<1$, where $\hat{A}$ is the invading phenotype. During the following l generations of the environmental cycle, the phenotype favoured by success bias is switched, such that $\frac{\beta \left(A\right)}{\beta \left(\hat{A}\right)}>1$. We then proceed to find expressions for the expectation and variance of the change in the frequency of phenotype $\hat{A}$ after n=k+l generations. The proof is in appendix C.

**Drift and diffusion terms in a changing environment.** Let x be the initial frequency of the invading phenotype and $X_{t}$ the number of individuals with that phenotype after n generations. Then,


(3.11) $$E\left[X_{n}/N-x\right]≃x\left(1-x\right)S_{n}/N_{e}\;\;\;\;\;\text{and}\;\;\;\;V\left(X_{n}/N-x\right)≃nx\left(1-x\right)/N_{e}\;,$$

where $S_{n}=\sum_{t=1}^{n}N(1-\beta_{t})$ and $\beta_{t}$ is β(A) at generation t.

Note that here, we have the ‘average selection coefficient’ during a cycle of n generations, $S_{n}/n$ as the selection coefficient in equation (3.8). Using the drift and diffusion terms and following Ram *et al*. [2], we can approximate the fixation probability in a changing environment.

***Result 3.6*** (*Fixation probability in a periodically changing environment*). *The fixation probability of an invading phenotype under periodical environmental changes is approximately*


(3.12) $$\overset{˜}{\pi }\left(x\right)=\frac{1-e^{-2\frac{S_{n}}{n}N_{e}x}}{1-e^{-2\frac{S_{n}}{n}N_{e}}}\;,$$

*where* x *is the initial frequency of the invading phenotype.*

**Figure 2.**
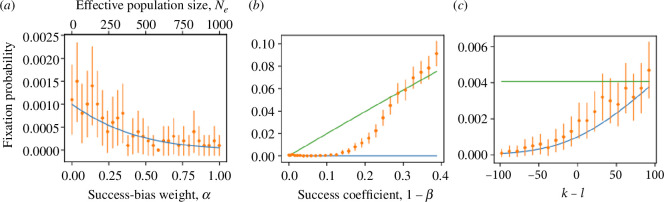
Fixation probability in a changing environment, k<l. (*a*) Fixation probability decreases with the success-bias weight (bottom *x*-axis) and effective population size (top *x*-axis). The approximation (blue; equation (3.12)) agrees with simulation results (orange). (*b*) Fixation probability increases with the success coefficient, β. When success bias is large (1−β>0.1), simulation results (orange) are underestimated by the changing environment approximation (blue; equation (3.12)). With even larger success bias (1−β>0.35), even the constant environment approximation (green; equation (3.8)) slightly underestimates simulation results, probably because the diffusion equation approximation assumes weak ‘selection’. (*c*) The approximation (blue) is robust to changes in environmental cycle length, as it agrees with simulations (orange) for different sizes of the changing environment cycle, where k and l are the number of generations each trait value is under success bias. When k>l, the approximation and the simulations are both very close to the constant environment approximation (green), because the more generations the rare phenotype is favoured, the more similar it is to the constant environment model, where it is always favoured by the success bias. Markers show an average of 10000 simulations, error bars show 75% (panels *a* and *c*) and 95% (panel *b*) confidence intervals. Refer to electronic supplementary material, figure S7 for the scenario where k>l. Here, population size is N=1000; panel (*a*): A=0.9, $\hat{A}=1$ (1−β=s=0.005), k=20 and l=80; panel (*b*): 0.01<A<0.99 and $\hat{A}=1$, which determines 1−β through $\beta =\beta (A)/\beta (\hat{A})$ and equation (2.5), k=20, l=80 and α=0.1; panel (*c*): A=0.8, $\hat{A}=1$ (1−β=s=0.0198) and α=0.1.

Importantly, the average selection coefficient, $S_{n}/n$, has the same sign as k−l. Therefore, when k>l, the fixation probability will increase with the success-bias weight α (similar to a constant environment, figure 1*a*), and when k<l, the fixation probability will decrease with the success-bias weight α (figure 2*a*). Furthermore, the fixation probability increases with the success coefficient (1−β, figure 2*b*; see below for how simulation results compare with the constant environment and changing environment approximations) and becomes larger as k−l increases, i.e. as the number of generations in which the invading phenotype is favoured increases (figure 2*c*).

**Numerical validation.** To validate the approximation for the fixation probability in a changing environment (equation (3.12)), we compare it with the results of simulations that use the DMD approximation (corollary 2). We find that the approximation fits the simulation results well for variable success-bias weights, α, which corresponds to the effective population size (figure 2*a*). However, the approximation is more sensitive to the value of the success-bias coefficient β (figure 2*b*). When the success coefficient 1−β is large, the approximation can break, as the fixation time can be lower than the number of generations in the cycle, n (refer to figure 1*d*), and therefore the average selection coefficient, $S_{n}/n$ is not a good estimate of the effective selection coefficient. We also changed the ratio between the number of cycles where $\hat{A}$ is favoured and disfavoured. We found that the approximation fits well regardless of the ratio, and that for a large k−l difference (with a constant cycle length, n=k+l=100), the changing environment approximation (equation (3.12)) converges to the constant environment approximation (equation (3.8); figure 2*c*). This makes sense as a constant environment can be viewed as an environment in which the cycle length is longer than the fixation time.

### 3.4. Optimal success-bias weight

In results 2–6, we assumed that the bias weight α is homogeneous in the population and constant, that is, it does not depend on any specific context. However, rational individuals could potentially adjust their bias weight to balance between success and prestige bias depending on their context, such as the number of individuals who have already chosen a role-model. Therefore, we examined what happens in the continuous-trait model if the ith copier evaluates its own optimal success-bias weight, $\alpha_{i}^{*}$, which minimizes the expected squared error between the chosen trait value and the ‘successful’ trait value $\hat{A}$,


(3.13) $$\alpha_{i}^{*}=argmin\sum_{j=1}^{N}\frac{\alpha A_{j}+(1-\alpha )K_{i-1,j}}{\sum_{l=1}^{N}\alpha A_{l}+(1-\alpha )K_{i-1,l}}{\left(\hat{A}-A_{j}\right)}^{2}\;,$$

where $A_{j}$ is the trait of role-model j and $K_{i-1,j}$ is the number of copiers that already chose role-model j by the time the ith copier chooses a role-model. Simply put, each copier i estimates what success-bias weight $\alpha_{i}^{*}$ will result in copying a trait that is most similar to the ‘successful’ trait value $\hat{A}$. Indeed, if the trait value is correlated with fitness, the optimal success-bias weight would increase the fitness of individuals. However, here we ignore the effects of natural selection, focusing instead on selectively neutral traits.

**Figure 3.**
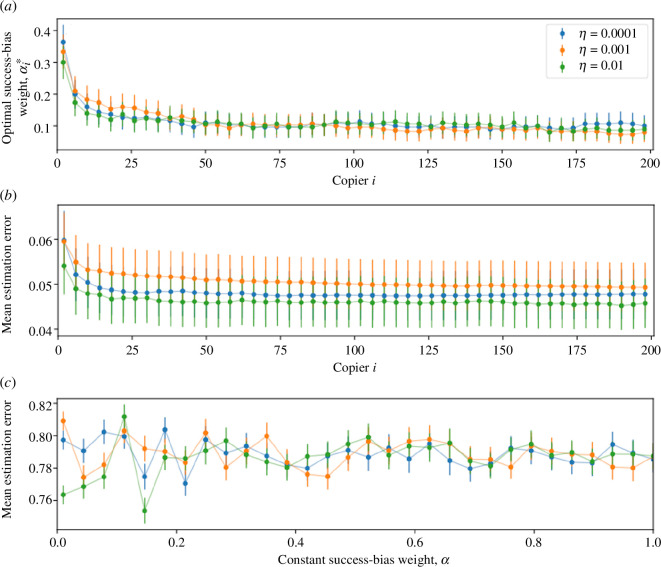
Advantage of an optimal success-bias weight. Both success-bias weight *α* (*a*) and estimation error (*b*) decrease during the role-model choosing process (within a single generation), demonstrating that prestige becomes more favoured by copiers as more copiers have made their choice. However, when *α* is homogeneous (*c*), the mean estimation error does not decrease, regardless of *α* or η. The mean estimation error in the homogeneous *α* model is larger by a factor of 10 than the optimal *α* model. Here, the *x*-axis is the index of the choosing copier, population size N=200; estimation error is normally distributed $e\sim N(0,\eta^{2})$ with standard deviation η=0.0001 (blue), 0.001 (orange), 0.01 (green), markers are the average of 300 simulations.

We find that when copiers choose their success-bias weight according to equation (3.13), it quickly decreases with the number of copiers that have already chosen a role-model and then stays at what appears to be an equilibrium (figure 3). Moreover, the estimation error is much lower compared with a constant success-bias weight, which gives roughly the same high estimation error to all copiers (compare figure 3*b* with 3*c*): in this example, the optimal success-bias weight gives an estimation error (difference between the chosen and ‘successful’ trait) that converges to 0.046, whereas a constant success-bias weight gives values greater than 0.74.

## 4. Discussion

Some cultural traits or cultural role-models may be copied more often than others owing to transmission biases. One such bias is success bias, in which copiers are more likely to copy a successful role-model. It has been suggested that because it is hard to estimate success, a more common bias is a bias towards role-models perceived to be successful. This perceived success can be determined by performance with respect to another trait, i.e. indirect success [4,9], or by ‘the amount of voluntary deference and attention received by models’ [8], i.e. prestige [18–20] (but refer to Chellappoo [37] for a critical examination of the concept of prestige).

We developed two cultural evolutionary models, one with continuous and one with dichotomous trait values. Our models include both indirect success and prestige biases, where the latter is a bias towards role-models with many copiers. We model these biases using a stochastic role-model choice process: each copier, in turn, randomly chooses a role-model, and this choice is affected both by the estimated success of each potential role-model and the number of copiers that already chose each role-model (equation (2.10)). Hence, our models have two ‘nested’ stochastic processes: the role-model choice process within each generation, and the cultural evolutionary process between generations. To simplify the mathematical and computational analysis, we developed analytic approximations for the role-model choice process using the GBD (result 1) and the DMD (corollary 2). The latter is especially useful, as it approximates the entire role-model choice process and only requires us to assume that the relative effect of success and prestige is a characteristic of the role-model and not the copier.

Analysing the dichotomous-trait model using the DMD approximation, we found approximations for the fixation probability and fixation time of a cultural trait under biased transmission in a constant environment. Our approximations are similar to Kimura’s evolutionary-genetic approximations, in that (i) the strength of success bias towards the invading cultural trait, $\beta =\beta (\hat{A})/\beta (A)$, is equivalent to the selection coefficient in favour of a beneficial allele, s; and (ii) decreasing the relative weight of success versus prestige bias, α, decreases the effective population size, $N_{e}$. Therefore, when either α or 1−β increases, the fixation probability increases (figure 1*a*,*b*). However, while increasing the s=1−β decreases the fixation time, as ‘selection’ is stronger (figure 1*d*), increasing the success-bias weight α increases the conditional fixation time (figure 1*c*). This is because, when the invading phenotype manages to fix in a population with strong prestige bias, it will do so faster compared with a population with weak prestige bias, as strong prestige leads to a *rich-getting-richer* process.

We also analysed the dichotomous-trait model in a periodically changing environment in which the identity of the success-biased trait switches after a fixed amount of generations (figure 2). We again derive an approximation for the fixation probability, which works well when the success coefficient 1−β is low. In the case of a changing environment, two key values are the number of generations k and l in which the invading and resident traits are favoured by success bias, respectively. When k>l, strong success (high α) will increase the fixation probability (electronic supplementary material, figure S7), but when k<l, strong prestige (low α) will increase the fixation probability (figure 2*a*). This is because prestige accelerates the evolutionary dynamics, which allows the invading trait to fix before the environment changes to favour the resident trait. In all cases, increasing the success coefficient 1−β, which is equivalent to increasing the strength of selection, will increase the fixation probability (figure 2*b*).

Finally, we examined a scenario in which copiers can adjust their success-bias weight, α, to minimize their copying error, i.e. copy trait values closer to the optimal value. We found that as the role-model choice process proceeds (i.e. more copiers make their choices), both the success-bias weight (adjusted by copiers) and the estimation error decrease. The latter is significantly lower than in a population using a constant, fixed success-bias weight, regardless of the value of the constant weight (figure 3). This suggests that the later a copier makes its choice, the more it should rely on the choices of previous copiers (prestige), and the less it should rely on its own estimation of the success of role-models. The rationale, then, is that the more information a copier has, e.g. by using others as information sources, the more informative and effective his choice can be.

Chudek *et al*. [19] report the first direct tests in children that suggest the existence of prestige bias, defined as the tendency to learn from individuals to whom others have preferentially attended, learned or deferred. Their definition of prestige is similar to ours. They showed that the odds of 3–4-year-old children learning from an adult role-model to whom bystanders had previously preferentially attended for 10 s were more than twice those of learning from a role-model whom bystanders ignored. They also note that prestige effects are domain sensitive: they found that prestigious role-models were attended more when demonstrating artefact use, whereas role-models presenting food preferences had less attendants, suggesting that the domain itself (artefact use versus food preference) can affect the attendance, and hence the prestige of the role-model. This led to the suggestion that when the trait is costly to learn individually, prestige will have a stronger bias [19]. It would be interesting to include costs in our model to try and observe these effects and dynamics in a large population.

According to Henrich & Broesch [4], natural selection has favoured the emergence of psychological biases for learning from those individuals most likely to possess adaptive information. The authors studied Fijian villages to examine if and how such biases emerge in a small-scale society. They found that Fijian villagers are more likely to learn from role-models perceived as more successful/knowledgeable, both within and across domains. Their research thus suggests that copying from those perceived as successful, rather than those who are actually successful, is a common phenomenon. They show that the social networks representing copier–role-model relationships are centralized, which is consistent with the prediction that people substantially share notions about who is a good cultural model, but that individuals’ role-model selections are influenced by multiple factors.

Dunbar [38] hypothesized that larger, more complex brains can store and manage more information and, in turn, this information can support the costs of a larger brain. Following this, Muthukrishna & Henrich [39] suggested that prestige can directly affect human physical evolution. They present a concept called *cultural brains*—brains that evolved primarily for the acquisition of adaptive knowledge. They then develop a model that predicts a strong relationship between brain size and group size, because group size also provides access to more adaptive knowledge. They also presented the *cumulative cultural brain* hypothesis, which proposes that human brains have evolved with an ability and tendency for selective, high-fidelity social learning. As part of this process, there are a variety of strategies and biases that have evolved to hone in on the most adaptive knowledge. These strategies and biases include direct and indirect cues of the popularity of cultural traits (e.g. success and prestige biases). They suggest that one of the reasons for the extreme increase in brain size in humans is the ability to ‘cheaply’ acquire adaptive knowledge through transmission biases such as prestige.

Prestige bias can help to cheaply estimate and acquire knowledge, which may facilitate survival and reproduction. However, it is not always the case, and there could be negative repercussions to this bias, such as invasion of maladaptive traits. Takahashi & Ihara [40] mention that social learning not only takes the form of random copying of other individuals, but also involves learners’ choice of what to learn and from whom to learn. They suggest a best-of-*k* model where an individual samples k role-models and chooses the one he deems most ‘successful’. They mention that a previous mathematical analysis has shown that it may sometimes result in maladaptive cultural evolution when the pay-offs associated with cultural variants vary stochastically. In such a case, learners may be selectively disfavoured and in the long run replaced by unbiased learners, who simply copy someone chosen at random. They developed new mathematical models that are simpler and mathematically tractable. They found that best-of-*k* learning, unlike unbiased learning, can facilitate the invasion of an on average inferior variant that sometimes gives a very high pay-off (refer to [21] and references therein). Our model, which includes both success and prestige bias, is consistent with this claim. When a maladaptive trait is ‘piggybacking’ on a role-model with high influence (joint effect of success and prestige), the former could spread in the population. In addition, best-of-*k* learning can be stable against invasion by unbiased learning if social learning is sometimes combined with individual learning [40]. Our model includes only social learning, and not individual learning, but it could be interesting to combine it with individual learning and see how it affects the dynamics.

Prestige bias can also accelerate the reversal of harmful traditions such as child marriage and domestic violence. Efferson *et al*. [41] suggest a *spillover* mechanism, in which an intervention affects a large enough group in a target population, so that others not included in the intervention also change their behaviour. They find that there are individuals who act as *agents*, who are often observed, and therefore they are ideal targets for interventions. This is similar to prestigious role-models in our model, which are copied more often, and will therefore spread their trait faster and wider in the population. They also suggest a way to use this phenomenon to change existing traditions in a population. It is very clear, however, that just as it can be used to end harmful traditions, the same agents could start harmful traditions.

Others have analysed models with interactions between different transmission biases. Hong [42] studied a model with both conformity and success bias (which he calls ‘pay-off bias’). He showed that an intermediate level of conformity bias—not too little but not too much—can be adaptive and evolve to prevent the invasion of low-success traits while allowing the invasion of high-success traits (for another example of adaptive filtering, refer to [43]). Similar to our model (equation (2.10)), Hong [42, eqn. 1] also additively combined the two transmission biases. However, transmission biases can be combined in many ways. For example, Denton *et al*. [11, eqn. 1] combined frequency-dependent bias and genetically determined content bias multiplicatively. Ammar *et al*. [44] studied a model in which individuals have a repertoire of cultural variants to choose from, and both variant choice and transmission through social learning are success biased. Moreover, they also included the possibility to ‘forget’ infrequently used variants; therefore, because usage is success biased, memory is also success biased. It remains to be seen how different assumptions on the mechanisms of learning and forgetting affect the evolutionary dynamics under different and interacting transmission biases.

One path forward is an analysis of the dynamics of the optimal success-bias weight model, in which every copier chooses its α. It would be interesting to see if the mean estimation error and the varying weight, $\alpha^{*}$, converge to specific values and how they are affected by the model parameters. It may also be possible to relax the assumptions required for our approximations, such as homogeneous estimation error and success-bias weight. Another possibility is to model prestige bias in a different way. For example, using a Moran model [45], one could build a model with overlapping generations, which would mix the within-generation model role-model choice process and the between-generation evolutionary dynamics. Finally, it would be interesting to analyse the fixation probability and time in the continuous model and determine how the results compare with those from the dichotomous model.

Another way to expand our model is to account for the two types of prestige or leadership suggested by Van Vugt & Smith [46] that are attributed to Confucius and Machiavelli. Confucius viewed leaders as role-models who exercise influence through possessing superior knowledge, skills and (outstanding) personal qualities. This fits the success bias in our model. In contrast, Machiavelli viewed leaders as rulers who exercise influence by imposing costs through (the threat of) punishment and violence. Van Vugt & Smith suggest that these opposing views are both partially supported by the available evidence but each one on its own offers an incomplete view of the complex and dynamic concept of leadership. Henrich & Gil-White [18] have suggested a similar distinction between ‘prestige’ and ‘dominance’ or between ‘persuasion’ and ‘force’. Several adjustments could be made so that our model reflects these leadership styles, such as assuming there is a correlation between phenotype and leadership style. The resulting cultural evolutionary dynamics and their dependence on the costs and benefits are intriguing.

## 5. Conclusions

We studied a model of cultural evolution under two transmission biases: the commonly studied success bias, together with prestige bias, which has so far received less attention from modellers. We found approximations for these complex dynamics. We then showed that success bias affects the evolutionary dynamics much like natural selection does, whereas prestige bias has a similar effect to random genetic drift. We also find a clear advantage to individuals that can choose the relative weight of the two biases.

## Data Availability

Source code is available at GitHub [47] and deposited on Zenodo [31]. Supplementary material is available online [48].

## Appendix A. General binomial distribution approximation

**Proving** $E[K_{Nj}]=\alpha_{j}\cdot \beta (A_{j}+e)/\bar{\alpha \cdot \beta (A+e)}$**, where the average in the denominator is over the role-models index,** j.

*Proof.* The initial influence of role-model j based on equation (2.10) is

(A 1)
$$G_{1,j}=\frac{\alpha_{j}\cdot \beta (A_{j}+e)}{\sum_{m=1}^{N}\alpha_{m}\cdot \beta (A_{m}+e)}\;.$$

The denominator of equation (A 1) can also be formulated as:

(A 2)
$$\sum_{m=1}^{N}\alpha_{m}\beta (A_{m}+e)=N\cdot \bar{\alpha \cdot \beta (A+e)}\;,$$

where $\bar{\alpha \beta (A+e)}$ is the mean value of $\alpha_{m}\cdot \beta (A_{m}+e)$. Using equation (A 2) and corollary 1 we get,

(A 3)
$$E\left[K_{N,j}\right]=\alpha_{j}\cdot \beta \left(A_{j}+e\right)/\bar{\alpha \cdot \beta \left(A+e\right)}\;.$$

## Appendix B. Drift and diffusion in a constant environment

**Proving drift and diffusion terms in a constant environment.** Let x and $x^{\prime }$ be the frequency of type $\hat{A}$ in a population with N individuals in the current and next generation, and β be the success coefficient of phenotype A, $\beta =\beta \left(A\right)<\beta \left(\hat{A}\right)=1$. Then,

$$E[x^{\prime }-x]\approx x(1-x)(1-\beta )\;,\;\;\;\;V(x^{\prime }-x)\approx x(1-x)\left(\frac{1}{\alpha N+(1-\alpha )}\right)\;.$$

*Proof.* Let X be the number of individuals of type $\hat{A}$ such that x=X/N. $X^{\prime }$ is the number of individuals with $\hat{A}$ in the next generation. The expected number of individuals is (owing to the DMD approximation),

(B 1)
$$E[X^{\prime }]=N\frac{\alpha_{1}}{\alpha_{1}+\alpha_{2}}\;,$$

where $\alpha_{1}=\alpha^{\prime }X$ and $\alpha_{2}=\alpha^{\prime }(N-X)\beta$, from equation (2.11). To use frequencies instead of counts, $E[x^{\prime }]=E[X^{\prime }/N]=\frac{1}{N}E[X^{\prime }]$. Putting it together,

(B 2)
$$\begin{array}{ll} E\left[x^{\prime }\right] & =\frac{1}{N}N\frac{\alpha^{\prime }xN}{\alpha^{\prime }xN+\alpha^{\prime }N\left(1-x\right)\beta }=\frac{x}{x+\left(1-x\right)\beta }\\ & =\frac{x}{x+\left(1-x\right)-\left(1-x\right)+\left(1-x\right)\beta }=x\frac{1}{1-\left(1-x\right)\left(1-\beta \right)}\\ & =x\left(1+\left(1-x\right)\left(1-\beta \right)+o\left(1-\beta \right)\right)=x+x\left(1-x\right)\left(1-\beta \right)+o\left(1-\beta \right)\;, \end{array}$$

following Durrett [49, p. 253, ch. 7.2] and because $1/(1-y)=1+y+y^{2}+\ldots$.

We therefore have

(B 3)
$$E[x^{\prime }-x]=E[x^{\prime }]-E[x]=x(1-x)(1-\beta )+o(1-\beta )\;,$$

which gives us the drift term of the diffusion equation.

Using the variance of the DMD,

(B 4)
$$V(X^{\prime })=N\frac{\alpha_{1}}{\alpha_{1}+\alpha_{2}}\left(1-\frac{\alpha_{1}}{\alpha_{1}+\alpha_{2}}\right)\left(\frac{N+\alpha_{1}+\alpha_{2}}{1+\alpha_{1}+\alpha_{2}}\right)\;.$$

Again, we want to use frequencies so we have $V(X^{\prime }/N)=\frac{1}{N^{2}}V(x^{\prime })$. Putting it together with our model notations,

(B 5)
$$V(x^{\prime })=\frac{1}{N^{2}}N\frac{x}{x+(1-x)\beta }\left(1-\frac{x}{x+(1-x)\beta }\right)\left(\frac{N+\alpha^{\prime }xN+\alpha^{\prime }N(1-x)\beta }{1+\alpha^{\prime }xN+\alpha^{\prime }N(1-x)\beta }\right)\;.$$

Following Durrett [49, ch. 7.2], we assume β≈1, such that

(B 6)
$$\frac{x}{x+(1-x)\beta }\approx x\;$$

and for the entire variance expression we get

(B 7)
$$V(x^{\prime })\approx \frac{1}{N}x(1-x)\left(\frac{N+\alpha^{\prime }xN+\alpha^{\prime }N-\alpha^{\prime }xN}{1+\alpha^{\prime }xN+\alpha^{\prime }N-\alpha^{\prime }xN}\right)=x(1-x)\left(\frac{1+\alpha^{\prime }}{1+\alpha^{\prime }N}\right)\;.$$

The current frequency x is a given, such that V(x)=0, and therefore

(B 8)
$$V(x^{\prime }-x)=V(x^{\prime })-V(x)\approx x(1-x)(\frac{1+\alpha^{\prime }}{1+\alpha^{\prime }N})\;.$$

$\alpha^{\prime }$ is the odds ratio of the bias weight,

(B 9)
$$\alpha^{\prime }=\frac{\alpha }{1-\alpha }\;.$$

Combining equations (B 8) and (B 9) we get

(B 10)
$$V(x^{\prime }-x)\approx x(1-x)\left(\frac{1+\frac{\alpha }{1-\alpha }}{1+\frac{\alpha }{1-\alpha }N}\right)=x(1-x)(\frac{1}{\alpha N+(1-\alpha )})\;.$$

This gives the diffusion term of the diffusion equation.

## Appendix C. Drift and diffusion in a changing environment

**Proving drift and diffusion terms in a changing environment.** Let x be the initial frequency of the invading phenotype and $X_{t}$ is the number of individuals with the phenotype at time t. Then,

$$E\left[X_{t}/N-x\right]≃x\left(1-x\right)S_{t}/N_{e}\;\;\;\;\;\text{and}\;\;\;\;V\left(X_{t}/N-x\right)≃tx\left(1-x\right)/N_{e}\;,$$

where $S_{t}=\sum_{i=1}^{t}N\left(1-\beta_{t}\right).$

*Proof.* Let $s_{t}=N(1-\beta_{t})$ and $S_{n}=\sum_{i=1}^{n}s_{i}$, where $\beta_{t}$ is β(A) at generation t. We prove by induction both terms in equation (3.11). From equation (B 3) we know that

(C 1)
$$E\left[\frac{X_{t+1}}{N}-\frac{X_{t}}{N}|X_{t}\right]=\frac{X_{t}}{N}\left(1-\frac{X_{t}}{N}\right)(1-\beta_{t+1})=\frac{1}{N}\frac{X_{t}}{N}\left(1-\frac{X_{t}}{N}\right)s_{t+1}\;.$$

Also note that using the definition of $V(y)=E[y^{2}]-{\left(E[y]\right)}^{2}$

(C 2)
$$\begin{array}{ll} E\left[\frac{X_{t}}{N}\left(1-\frac{X_{t}}{N}\right)\right] & =E\left[\frac{X_{t}}{N}-{\left(\frac{X_{t}}{N}\right)}^{2}\right]=E\left[\frac{X_{t}}{N}\right]-E\left[{\left(\frac{X_{t}}{N}\right)}^{2}\right]\\ & =E\left[\frac{X_{t}}{N}\right]-V\left(\frac{X_{t}}{N}\right)-{\left(E\left[\frac{X_{t}}{N}\right]\right)}^{2}\;. \end{array}$$

We can now use the induction assumption of $V\left(\frac{X_{t}}{N}\right)$ to get

(C 3)
$$E\left[\frac{X_{t}}{N}\left(1-\frac{X_{t}}{N}\right)\right]≃E\left[\frac{X_{t}}{N}\right]\left(1-E\left[\frac{X_{t}}{N}\right]\right)-\frac{1}{N_{e}}tx(1-x)\;.$$

From equation (C 1) we know that

(C 4)
$$\begin{array}{ll} E\left[\frac{X_{t+1}}{N}-\frac{X_{t}}{N}\right] & =\frac{1}{N}s_{t+1}E\left[\frac{X_{t}}{N}\left(1-\frac{X_{t}}{N}\right)\right]≃\frac{1}{N}s_{t+1}\left(E\left[\frac{X_{t}}{N}\right]\left(1-E\left[\frac{X_{t}}{N}\right]\right)-\frac{1}{N_{e}}tx\left(1-x\right)\right)\\ & ≃\frac{1}{N}s_{t+1}\cdot E\left[\frac{X_{t}}{N}\right]\left(1-E\left[\frac{X_{t}}{N}\right]\right)-\frac{1}{N_{e}N}s_{t+1}tx\left(1-x\right)\;. \end{array}$$

Now we omit $O\left(\frac{1}{Ne\cdot N}\right)$ and get

(C 5)
$$E\left[\frac{X_{t+1}}{N}-\frac{X_{t}}{N}\right]≃\frac{1}{N}s_{t+1}\cdot E\left[\frac{X_{t}}{N}\right]\left(1-E\left[\frac{X_{t}}{N}\right]\right)\;.$$

We now look at the induction assumption to see that

(C 6)
$$E\left[\frac{X_{t}}{N}-x\right]=E\left[\frac{X_{t}}{N}\right]-E[x]=E\left[\frac{X_{t}}{N}\right]-x\;,$$

so using the assumption we get

(C 7)
$$\begin{array}{c} E\left[\frac{X_{t}}{N}\right]≃\frac{1}{N}S_{t}x(1-x)+x\;,\\ 1-E\left[\frac{X_{t}}{N}\right]≃1-\frac{1}{N}S_{t}x(1-x)+x\;. \end{array}$$

We use both expressions in equation (C 5) and get

(C 8)
$$\begin{array}{ll} E\left[\frac{X_{t+1}}{N}-\frac{X_{t}}{N}\right] & ≃\frac{1}{N}s_{t+1}\left(\frac{1}{N}S_{t}x\left(1-x\right)+x\right)\left(1-\frac{1}{N}S_{t}x\left(1-x\right)+x\right)\\ & ≃\frac{1}{N}s_{t+1}\cdot x\left(1-x\right), \end{array}$$

after again omitting $O\left(\frac{1}{N^{2}}\right)$ terms. To conclude the proof, we note that

(C 9)
$$E\left[\frac{X_{t+1}}{N}-x\right]=E\left[\frac{X_{t+1}}{N}-\frac{X_{t}}{N}\right]+E\left[\frac{X_{t}}{N}-x\right]\;,$$

so again using the induction assumption, together with equation (C 8) we get

(C 10)
$$\begin{array}{c} E\left[\frac{X_{t+1}}{N}-x\right]≃\frac{1}{N}s_{t+1}\cdot x(1-x)+\frac{1}{N}S_{t}\cdot x(1-x)\\ ≃\frac{1}{N}x(1-x)(S_{t}+s_{t+1})≃\frac{1}{N}S_{t+1}x(1-x)\;, \end{array}$$

which proves the drift term.

For the diffusion term, we use a property of variance,

(C 11)
$$V\left(\frac{X_{t+1}}{N}\right)=E\left[V\left(\frac{X_{t+1}}{N}|X_{t}\right)\right]+V\left(E\left[\frac{X_{t+1}}{N}|X_{t}\right]\right)\;.$$

Using equation (C 1) we see that

(C 12)
$$\begin{array}{l} E\left[\frac{X_{t+1}}{N}|X_{t}\right]-E\left[\frac{X_{t}}{N}|X_{t}\right]=\frac{1}{N}s_{t+1}\frac{X_{t}}{N}\left(1-\frac{X_{t}}{N}\right)\\ E\left[\frac{X_{t+1}}{N}|X_{t}\right]=\frac{X_{t}}{N}+\frac{1}{N}s_{t+1}\frac{X_{t}}{N}\left(1-\frac{X_{t}}{N}\right)\;. \end{array}$$

Using equation (B 10) we get

(C 13)
$$V\left(\frac{X_{t+1}}{N}|X_{t}\right)=\frac{1}{N_{e}}\frac{X_{t}}{N}\left(1-\frac{X_{t}}{N}\right)\;,$$

and using the equation $y^{\prime }(1-y^{\prime })≃y(1-y)$ on the first part of equation (C 11) we get

(C 14)
$$E\left[V\left(\frac{X_{t+1}}{N}|X_{t}\right)\right]=\frac{1}{N_{e}}E\left[\frac{X_{t}}{N}\left(1-\frac{X_{t}}{N}\right)\right]≃\frac{1}{N_{e}}x(1-x)\;.$$

Moving on to simplify the second part of equation (C 11) using equation (C 12),

(C 15)
$$V\left(E\left[\frac{X_{t+1}}{N}|X_{t}\right]\right)=V\left(\frac{X_{t}}{N}+\frac{1}{N}s_{t+1}\frac{X_{t}}{N}\left(1-\frac{X_{t}}{N}\;\right)\right).$$

Now, because $\frac{X_{t}}{N}$ is a frequency, i.e $0\leq X_{t}/N\leq 1$, we know that $V\left(\frac{X_{t}}{N}\left(1-\frac{X_{t}}{N}\right)\right)\leq \frac{1}{4}$. We therefore find that

(C 16)
$$V\left(\frac{1}{N}s_{t+1}\frac{X_{t}}{N}\left(1-\frac{X_{t}}{N}\right)\right)\leq \frac{1}{4N^{2}}s_{t+1}^{2},$$

and so it can be ignored. Combining our equations we get

(C 17)
$$V\left(E\left[\frac{X_{t+1}}{N}|X_{t}\right]\right)=V\left(\frac{X_{t}}{N}\right)+O\left(\frac{1}{N^{2}}\right)≃V\left(\frac{X_{t}}{N}\right)\;.$$

Using the induction assumption and equation (C 14),

(C 18)
$$V\left(\frac{X_{t+1}}{N}\right)≃\frac{1}{N_{e}}x\left(1-x\right)+\frac{1}{N_{e}}tx\left(1-x\right)≃\frac{1}{N_{e}}x\left(1-x\right)\left(t+1\right)\;,$$

which proves the diffusion term.

## References

[1] Cavalli-Sforza LL, Feldman MW. 1981 Cultural transmission and evolution: a quantitative approach, p. 16. Princeton, NJ: Princeton University Press. (10.1515/9780691209357) 7300842

[2] Ram Y, Liberman U, Feldman MW. 2018 Evolution of vertical and oblique transmission under fluctuating selection. Proc. Natl Acad. Sci. USA **115**, E1174–E1183. (10.1073/pnas.1719171115) 29363602 PMC5819448

[3] Lehmann L, Feldman MW. 2009 Coevolution of adaptive technology, maladaptive culture and population size in a producer-scrounger game. Proc. R. Soc. B **276**, 3853–3862. (10.1098/rspb.2009.0724) PMC2817275 19692409

[4] Henrich J, Broesch J. 2011 On the nature of cultural transmission networks: evidence from Fijian villages for adaptive learning biases. Phil. Trans. R. Soc. B **366**, 1139–1148. (10.1098/rstb.2010.0323) 21357236 PMC3049092

[5] Mesoudi A, O’Brien MJ. 2008 The cultural transmission of great basin projectile-point technology II: an agent-based computer simulation. Am. Antiq. **73**, 627–644. (10.1017/S0002731600047338)

[6] Borofsky T, Feldman MW. 2022 Success-biased social learning in a one-consumer, two-resource model. Theor. Popul. Biol. **146**, 29–35. (10.1016/j.tpb.2022.05.004) 35709950

[7] Rendell L *et al*. 2010 Why copy others? Insights from the social learning strategies tournament. Science **328**, 208–213. (10.1126/science.1184719) 20378813 PMC2989663

[8] Jiménez ÁV, Mesoudi A. 2019 Prestige-biased social learning: current evidence and outstanding questions. Palgrave Commun. **5**, 1. (10.1057/s41599-019-0228-7)

[9] Boyd R, Richerson PJ. 1985 Culture and the evolutionary process. Chicago, IL: University of Chicago Press.

[10] Molleman L, Pen I, Weissing FJ. 2013 Effects of conformism on the cultural evolution of social behaviour. PLoS One **8**, e68153. (10.1371/journal.pone.0068153) 23874528 PMC3707918

[11] Denton KK, Ram Y, Feldman MW. 2022 Conformity and content-biased cultural transmission in the evolution of altruism. Theor. Popul. Biol. **143**, 52–61. (10.1016/j.tpb.2021.10.004) 34793823

[12] Denton KK, Liberman U, Feldman MW. 2021 On randomly changing conformity bias in cultural transmission. Proc. Natl Acad. Sci. USA **118**, e2107204118. (10.1073/pnas.2107204118) 34417299 PMC8403876

[13] Denton KK, Ram Y, Liberman U, Feldman MW. 2020 Cultural evolution of conformity and anticonformity. Proc. Natl Acad. Sci. USA **117**, 13603–13614. (10.1073/pnas.2004102117) 32461360 PMC7306811

[14] Aljadeff N, Giraldeau LA, Lotem A. 2020 Competitive advantage of rare behaviours induces adaptive diversity rather than social conformity in skill learning. Proc. R. Soc. B **287**, 20201259. (10.1098/rspb.2020.1259) PMC7482281 32811312

[15] Kolodny O, Feldman MW, Lotem A, Ram Y. 2022 Differential application of cultural practices at the family and individual levels may alter heritability estimates. Behav. Brain Sci. **45**, e167. (10.1017/S0140525X21001576) 36098428

[16] Andersson M, Iwasa Y. 1996 Sexual selection. Trends Ecol. Evol. **11**, 53–58. (10.1016/0169-5347(96)81042-1) 21237761

[17] Cohen D, Lewin-Epstein O, Feldman MW, Ram Y. 2021 Non-vertical cultural transmission, assortment and the evolution of cooperation. Proc. R. Soc. B **288**, 20203162. (10.1098/rspb.2020.3162) PMC8150029 34034521

[18] Henrich J, Gil-White FJ. 2001 The evolution of prestige: freely conferred deference as a mechanism for enhancing the benefits of cultural transmission. Evol. Hum. Behav. **22**, 165–196. (10.1016/S1090-5138(00)00071-4) 11384884

[19] Chudek M, Heller S, Birch S, Henrich J. 2012 Prestige-biased cultural learning: bystander’s differential attention to potential models influences children’s learning. Evol. Hum. Behav. **33**, 46–56. (10.1016/j.evolhumbehav.2011.05.005)

[20] Nakata S, Masumi A, Toya G. 2024 Formalising prestige bias: differences between models with first-order and second-order cues. Evol. Hum. Sci. **6**, e21. (10.1017/ehs.2024.12) 38689894 PMC11058518

[21] Fogarty L, Wakano JY, Feldman MW, Aoki K. 2017 The driving forces of cultural complexity: Neanderthals, modern humans, and the question of population size. Hum. Nat. **28**, 39–52. (10.1007/s12110-016-9275-6) 27783325

[22] Anagnostopoulos A, Kumar R, Mahdian M. 2008 Influence and correlation in social networks. In Proceedings of the 14th ACM SIGKDD Int. Conf. on Knowledge Discovery and Data Mining*,* *Las Vegas, NV*. New York, NY: ACM. (10.1145/1401890.1401897)

[23] Peng S, Zhou Y, Cao L, Yu S, Niu J, Jia W. 2018 Influence analysis in social networks: a survey. J. Netw. Comput. Appl. **106**, 17–32. (10.1016/j.jnca.2018.01.005)

[24] Diga M, Kelleher T. 2009 Social media use, perceptions of decision-making power, and public relations roles. Public Relat. Rev. **35**, 440–442. (10.1016/j.pubrev.2009.07.003)

[25] Lee W, Xiong L, Hu C. 2012 The effect of Facebook users’ arousal and valence on intention to go to the festival: applying an extension of the technology acceptance model. Int. J. Hosp. Manag. **31**, 819–827. (10.1016/j.ijhm.2011.09.018)

[26] Kimura M. 1962 On the probability of fixation of mutant genes in a population. Genetics **47**, 713–719. (10.1093/genetics/47.6.713) 14456043 PMC1210364

[27] Kimura M, Ohta T. 1969 The average number of generations until fixation of a mutant gene in a finite population. Genetics **61**, 763–771. (10.1093/genetics/61.3.763) 17248440 PMC1212239

[28] Van Rossum V, Guido G. 2007 Python programming language. In USENIX. Ann. Tech. Conf, vol. **41**, p. 1,

[29] van der Walt S, Colbert SC, Varoquaux G. 2011 The numpy array: a structure for efficient numerical computation. Comput. Sci. Eng. **13**, 22–30. (10.1109/MCSE.2011.37)

[30] Hunter JD. 2007 Matplotlib: a 2D graphics environment. Comput. Sci. Eng. **9**, 90–95. (10.1109/MCSE.2007.55)

[31] Ram Y, Egozi S. 2024 Prestige bias in cultural evolutionary dynamics. Zenodo. (10.5281/zenodo.11424199) PMC11285825 39076362

[32] Denton KK, Ram Y, Feldman MW. 2023 Conditions that favour cumulative cultural evolution. Phil. Trans. R. Soc. B **378**, 20210400. (10.1098/rstb.2021.0400) 36688392 PMC9869458

[33] Drezner Z, Farnum N. 1993 A generalized binomial distribution. Commun. Stat. Theory. Meth. **22**, 3051–3063. (10.1080/03610929308831202)

[34] Frigyik BA, Kapila A, Gupta MR. 2010 introduction to the Dirichlet distribution and related processes. vol. 6. pp. 1–27. UWEETR-2010-0006. Seattle, WA: Department of Electrical Engineering, University of Washington.

[35] Durrett R. 1999 Essentials of stochastic processes. vol. 1. New York, NY: Springer.

[36] Slatkin M. 1981 Fixation probabilities and fixation times in a subdivided population. Evolution **35**, 477–488. (10.1111/j.1558-5646.1981.tb04911.x) 28563585

[37] Chellappoo A. 2021 Rethinking prestige bias. Synthese **198**, 8191–8212. (10.1007/s11229-020-02565-8)

[38] Dunbar RIM. 2009 The social brain hypothesis and its implications for social evolution. Ann. Hum. Biol. **36**, 562–572. (10.1080/03014460902960289) 19575315

[39] Muthukrishna M, Henrich J. 2016 Innovation in the collective brain. Philos. Trans. R. Soc. **371**, 20150192. (10.1098/rstb.2015.0192) PMC4780534 26926282

[40] Takahashi T, Ihara Y. 2019 Cultural and evolutionary dynamics with best-of-k learning when payoffs are uncertain. Theor. Popul. Biol. **128**, 27–38. (10.1016/j.tpb.2019.05.004) 31145878

[41] Efferson C, Vogt S, Fehr E. 2020 The promise and the peril of using social influence to reverse harmful traditions. Nat. Hum. Behav. **4**, 55–68. (10.1038/s41562-019-0768-2) 31792402

[42] Hong Z. 2022 Combining conformist and payoff bias in cultural evolution: an integrated model for human decision-making. Hum. Nat. **33**, 463–484. (10.1007/s12110-022-09435-x) 36515860

[43] Enquist M, Ghirlanda S. 2007 Evolution of social learning does not explain the origin of human cumulative culture. J. Theor. Biol. **246**, 129–135. (10.1016/j.jtbi.2006.12.022) 17275852

[44] Ammar M, Fogarty L, Kandler A. 2023 Social learning and memory. Proc. Natl Acad. Sci. USA **120**, e2310033120. (10.1073/pnas.2310033120) 37549253 PMC10433305

[45] Moran PAP. 1958 Random processes in genetics. Math. Proc. Camb. Philos. Soc. **54**, 60–71. (10.1017/S0305004100033193)

[46] Van Vugt M, Smith JE. 2019 A dual model of leadership and hierarchy: evolutionary synthesis. Trends Cogn. Sci. **23**, 952–967. (10.1016/j.tics.2019.09.004) 31629633

[47] Yoavram-lab. 2024 Prestigebias. GitHub. See https://github.com/yoavram-lab/PrestigeBias.

[48] Egozi S, Ram Y. 2024 Supplementary material from: Prestige bias in cultural evolutionary Dynamics. Figshare. (10.6084/m9.figshare.c.7303167) PMC11285825 39076362

[49] Durrett R. 2008 Probability models for DNA sequence evolution. vol. 2. New York, NY: Springer. (10.1007/978-0-387-78168-6)

